# Telehealth-Delivery of a Multicomponent Obesity Intervention in Older, Rural Adults With Obesity

**DOI:** 10.1093/geroni/igaa057.2289

**Published:** 2020-12-16

**Authors:** John Batsis, Curtis Petersen, Rima Al-Nimr, Tyler Gooding, Summer Cook, Todd Mackenzie

**Affiliations:** 1 Dartmouth College, Lebanon, New Hampshire, United States; 2 Dartmouth Hitchcock Medical Center, Lebanon, New Hampshire, United States; 3 University of New Hampshire, Durham, New Hampshire, United States

## Abstract

Older, rural residents with obesity aged ≥65 years have reduced access to health promotion programs due to geography. We conducted a 26-week intervention of 24 older obese adults (BMI≥30kg/m2) in a geographically isolated area in Northern New England. The telemedicine delivered intervention consisted of individual, weekly, dietitian visits focusing on caloric restriction, and twice-weekly physical therapist-led group strength training classes. Participants’ age was 73.4±4.4years (79% female); pre-post assessments consisted of bioelectrical impedance-based body composition, functional measures, and satisfaction questionnaires. Feasibility was high (50% enrolled, 85% completed). Weight decreased 4.5±3.8kg (4.5±0.5%; 48% achieving ≥5%), 30s sit-to-stand improved (+3.8±4.1repetitions), as did 6-minute walk, +76.2±70.1m (all p<0.001). Appendicular mass did not change (+0.20±2.3kg); % body and visceral fat both decreased (-1.8±2.8% [p=0.009], -1.2±2.7L [p=0.025]). Participants endorsed telemedicine (96%); 78% preferred a home-based study. Satisfaction was high (4.2/5) and only 17% faced difficulties. Despite geography, this intervention holds promise in improving physical function.

